# Impacts of stem cells from different sources on wound healing rate in diabetic foot ulcers: a systematic review and meta-analysis

**DOI:** 10.3389/fgene.2024.1541992

**Published:** 2025-01-28

**Authors:** Le Tong, Lin Tang, Bangli Tang, Jianna Zhang

**Affiliations:** ^1^ Department of Emergency Medicine, West China Hospital, Sichuan University/West China School of Nursing, Sichuan University, Chengdu, China; ^2^ Disaster Medical Center, Sichuan University, Chengdu, China; ^3^ Nursing Key Laboratory of Sichuan Province, Chengdu, China; ^4^ Department of Dermatology, Mianyang Central Hospital, School of Medicine, University of Electronic Science and Technology of China, Mianyang, China

**Keywords:** stem cells, different sources, diabetic foot ulcer, wound healing rate, meta-analysis

## Abstract

**Background:**

Diabetic foot ulcers (DFU) are a significant complication of diabetes, with huge implications on patient morbidity and healthcare costs. The objective of this meta-analysis was to evaluate the impacts of stem cells from different sources on wound healing rate in DFU patients.

**Methods:**

We systematically retrieved records via key databases PubMed, Cochrane Library, Web of Science, Embase, China National Knowledge Infrastructure (CNKI) and Wanfang from the inception to October 2024. The Stata 16.0 (Stata Corp, TX) software was used to perform the meta-analysis. Risk of bias in all included studies was evaluated by Cochrane Risk of Bias version 2.

**Results:**

A total of 24 studies involving 1,321 patients were included. There was an increased likelihood of wound healing with peripheral blood-derived stem cells, the most effective cells (odds ratios (OR) = 7.31, 95% CI: 2.90–18.47), followed by adipose-derived stem cells (OR = 5.23, 95% CI: 2.76–9.90), umbilical cord-derived stem cells (OR = 4.94, 95% CI: 0.61–40.03), bone-derived stem cells (OR = 4.36, 95% CI: 2.43–7.85) and other sources stem cells (OR = 3.16, 95% CI: 1.83–5.45). Nevertheless, only umbilical cord-derived stem cells showed statistical significance (p < 0.05). The heterogeneity ranged from non-existent in the adipose and peripheral blood groups (I^2^ = 0.00%) to moderate in the bone groups (I^2^ = 26.31%) and other groups (I^2^ = 30.62%), and substantial in the umbilical cord groups (I^2^ = 88.37%). Asymmetrical funnel plots pointed to publication bias, but the trim-and-fill method to correct for this brought the effect estimates even lower: based on the pooled OR, corrected OR was 3.40 (95% CI 2.39–4.84). Stem cell therapy was also associated with improvements in several secondary outcomes, suggesting its potential to influence the progression of DFU.

**Conclusion:**

Our study suggested that stem cells from different sources showed potential in promoting wound healing in DFU, although with some variation in effectiveness. Despite some publication bias and moderate heterogeneity, the overall therapeutic effect remained positive. These findings indicated that stem cell therapy might influence the progression of DFU.

## Introduction

Diabetic foot ulcers (DFU) refer to a condition in individuals who have been diagnosed with diabetes or have a history of the disease, characterized by infection, ulceration, or degradation of tissue in the foot. This condition frequently occurs alongside complications such as lower extremity neuropathy and/or peripheral arterial disease (Lipsky et al., 2020). Among the severe chronic complications of diabetes, DFU are particularly concerning. These ulcers adversely affect the physical health and significantly diminish the quality of life of affected individuals (Krasilnikova et al., 2022). Additionally, DFU lead to considerable psychological distress and financial strain for both the patients and their families, imposing a significant societal burden. Given its widespread impact, diabetic foot ulceration has become a critical public health issue (Edmonds et al., 2021). Statistics highlight the severity of this condition, showing that between 5% and 24% of patients with diabetic foot ulcers require amputation within 6–18 months after their initial diagnosis. Alarmingly, an amputation occurs approximately every 20 s among this population (Ibrahim, 2017). Moreover, about half of these patients die within 5 years following their amputation (Weledji and Fokam, 2014; Humphries et al., 2016).

Current therapeutic approaches for DFU include a variety of strategies: glycemic control, local wound debridement, changing wound dressings, applying antimicrobial treatments, vascular reconstruction, employing traditional Chinese medicinal compounds, hyperbaric oxygen therapy, and in severe cases, restoring lower limb blood perfusion (Singh et al., 2017; Chen et al., 2023). Despite these diverse interventions, the effectiveness of treatments for severe DFU is often limited, and some methods have inherent drawbacks. For example, extended use of high-dose antibiotics can disrupt normal bacterial balance, leading to secondary fungal infections and the development of drug-resistant bacteria (Liu et al., 2023a). Surgical options are also limited by technical challenges such as recanalization failures, vascular re-occlusions, and impaired microcirculation (Meloni et al., 2020).

Stem cell therapy holds significant promise in enhancing wound healing in DFU, largely due to its potential to regenerate tissue and modulate immune responses. Stem cells, particularly mesenchymal stem cells (MSCs), have been shown to promote wound healing by secreting growth factors and cytokines that accelerate tissue repair and reduce inflammation. For instance, studies have demonstrated that MSCs can improve angiogenesis, increase collagen synthesis, and recruit local and systemic cells involved in wound repair processes. Additionally, the immunomodulatory properties of stem cells help in mitigating the excessive inflammatory responses often seen in diabetic wounds, thereby preventing prolonged inflammation and further tissue damage (Sasaki et al., 2008; Tutuianu et al., 2021; Mimeault et al., 2007). Currently, stem cells used for the treatment of DFU are classified into autologous and allogeneic types, with primary sources including bone marrow, umbilical cord, adipose tissue, and placenta. However, the impact of stem cells from different sources on DFU healing rates has not been fully quantified (Yu et al., 2022).

This study aimed to explore the existing literature on stem cell therapies for DFU, assessing their efficacy in accelerating wound healing based on different cell sources. By providing a clearer understanding of the benefits and limitations of various stem cell therapies, this study seeked to inform clinical decision-making and highlight potential avenues for future research.

## Methods

We followed the Preferred Reporting Items for Systematic reviews and Meta-Analyses (PRISMA) checklist for reporting systematic reviews and meta-analysis. The study has been registered on the international prospective register of systematic reviews.

### Literature search

We conducted a thorough search to assess the association between cell therapy and ulcer healing. PubMed, Cochrane Library, Web of Science, Embase, China National Knowledge Infrastructure (CNKI) and Wanfang were searched from the inception to October 2024. The following search strategy was used: “stem cell,” or “progenitor cell,” or “mesenchymal stem cells,” or “adipose-derived,” or “bone marrow,” or “peripheral blood,” or “Umbilical cord,” or “mononuclear cell” paired with “diabetic” paired with “foot,” or “ulcer,” or “wound” (Supplementary Table S1).

### Study selection

The criteria for the inclusion of studies were pre-established as follows: 1) the study design must be either controlled clinical trials (CCTs); 2) the study population should consist of patients afflicted with diabetic foot ulcers; 3) the intervention group should have received some form of stem cells therapy, including but not limited to bone-derived stem cells, adipose-derived stem cells, or peripheral blood-derived stem cells; 4) the comparison group should have received either standard care or a placebo; 5) the studies must report on the outcome measure of the wound healing rate in patients; 6) the study should have a sample size of 10 or more participants.

Conversely, studies were excluded based on the following criteria: 1) studies that suffered from incomplete data with no possibility of contacting the authors for further information; 2) studies lacking a control group; 3) non-empirical studies such as letters, editorials, conference abstracts, case reports, reviews, and study protocols; 4) animal studies.

### Data extraction

Data extraction was performed by two independent reviewers utilizing standardized forms to ensure accuracy and reliability. Any discrepancies between reviewers were resolved through discussion until a consensus was achieved. The quality of the included studies was appraised based on the pre-specified inclusion criteria. The extracted data encompassed author (s), publication year, country, study design, sample size, demographic data of participants (age, gender), original ulcer size, cell source, donor type, intervention method, cell dose, follow-up time, and reported results.

### Quality assessment

The assessment of the risk of bias within the included studies was conducted independently by two reviewers using the Cochrane Collaboration’s risk of bias tool (RoB 2.0). This tool facilitated a systematic evaluation across five domains of bias, namely: bias arising from the randomization process; bias due to deviations from intended interventions; bias due to missing outcome data; bias in measurement of the outcome; and bias in the selection of the reported result. Studies were then categorized based on their risk of bias as low (meeting all or at least four of the low-risk criteria), having some concerns, or high risk, to ensure a rigorous analysis of the evidence.

### Statistical analysis

Statistical analyses were performed using the random-effects model, following the DerSimonian and Laird method, to accommodate inherent between-study variability. Pooled odds ratios (OR) and mean differences (MD) with 95% confidence intervals (CI) were calculated to assess the efficacy of stem cell therapies. Heterogeneity among the included studies was quantified using the I^2^ statistic, where values of 25%, 50%, and 75% were considered as low, moderate, and high heterogeneity, respectively. Heterogeneity was also assessed through visual inspection of L’Abbé plots. Moreover, sensitivity analysis was used to test the stability of the results, and publication bias was estimated using a funnel plot, trim-and-fill analysis and Egger’s and Begg’s tests, with a significance threshold of P < 0.05. Subgroup analyses were conducted based on donor source, study design, and follow-up duration to explore potential sources of variability in treatment effects. All statistical analyses were performed using STATA 16.0 software. Significance was set at a p-value of less than 0.05 for all tests.

## Results

### Literature search

**FIGURE 1:**
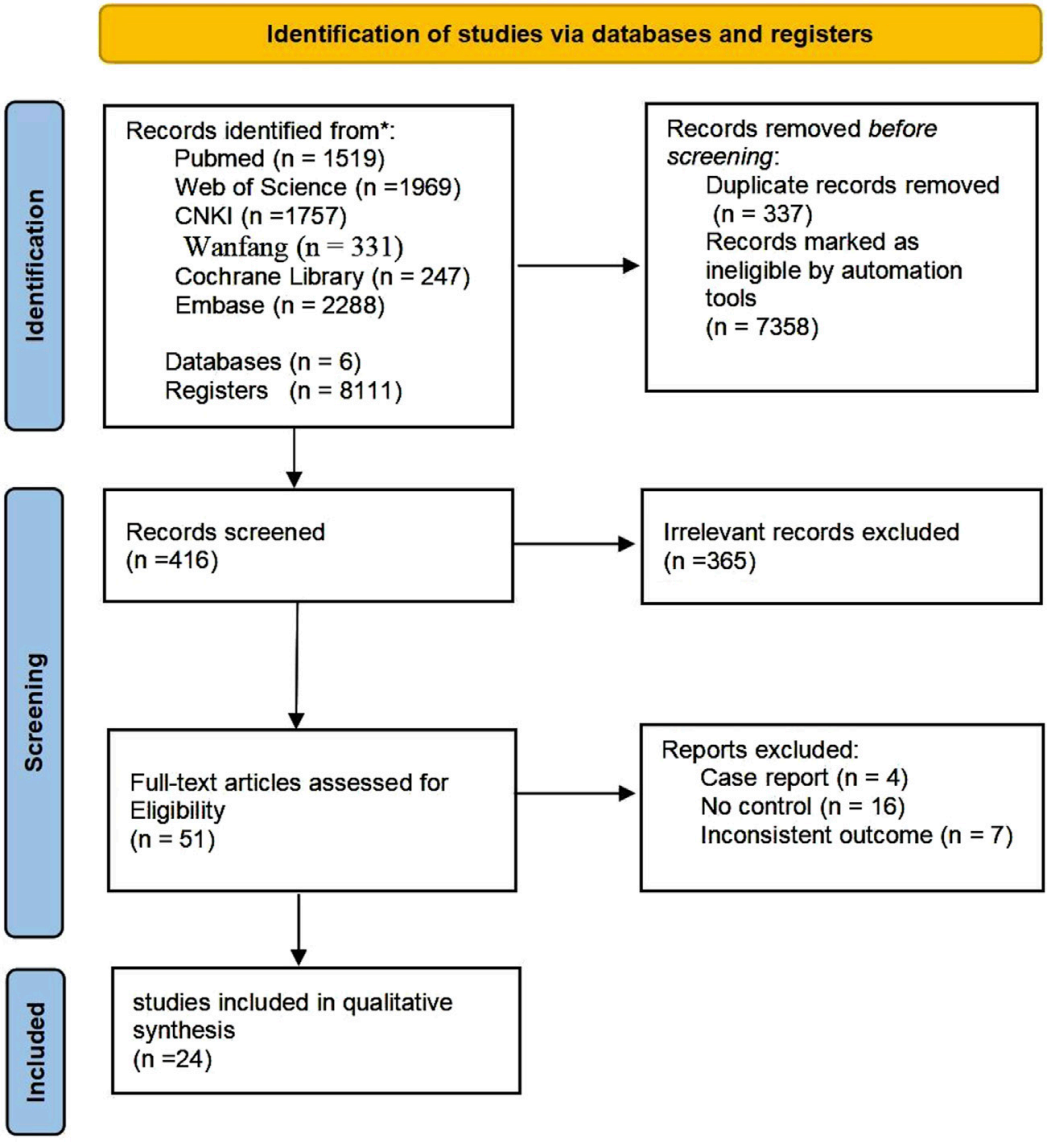
Flow diagram of study selection.

Initially, a comprehensive search across six databases and various registers yielded a total of 8,111 records. Of these, 337 were identified as duplicates and 7358 were automatically disqualified. Following a evaluation of titles and abstracts, 365 articles were excluded based on the inclusion and exclusion criteria. The remaining 51 full-text articles were assessed for eligibility, leading to the exclusion of an additional 98 reports due to various reasons such as non-controlled study designs, inconsistent outcomes, or because they were case reports or reviews. Ultimately, 24 studies met all inclusion criteria and were included in the qualitative synthesis of our meta-analysis (Huang et al., 2005; Zhang et al., 2007; Debin et al., 2008; Han et al., 2010; Jain et al., 2011; Lu et al., 2011; Ozturk et al., 2012; Kirana et al., 2012; You et al., 2012; Dubsky et al., 2013; Mohammadzadeh et al., 2013; Dubský et al., 2014; Lavery et al., 2014; He, 2014; Zhang et al., 2016; Park et al., 2018; Lonardi et al., 2019; Moon et al., 2019; Smith et al., 2020; Uzun et al., 2021; Liu et al., 2023b; Mrozikiewicz-Rakowska et al., 2023; Wang et al., 2024a; Wang et al., 2024b) (Figure 1).

### Characteristics of included articles

Table 1 showed the characteristics of included study. Most studies originated from Asia and Europe, with only one study from North America. Sample sizes varied widely, ranging from 12 to 167 patients, with ages between 56.6 and 64.1 years. Gender distribution differed across studies, with the total number of males slightly exceeding the total number of females. The average wound size ranged from 2.0 cm^2^ to 23.5 cm^2^. Stem cell sources included bone marrow, peripheral blood, fat, umbilical cord, and other tissues, with administration methods being either injection or application. The cell doses and follow-up periods varied, with the shortest follow-up being 30 days and the longest reaching 48 months.

**TABLE 1:** The baseline characteristics of the included studies.

Study	Country	Study design	Participant cases	Wound size (cm^2^)		Age		F/M	Cell type	Cell source	Method	Cell dose	Follow-up duration
				E	C	E	C						
Huang et al. (2005)	China	CCT	28	2.7 ± 1.3	2.4 ± 1.2	71.1 ± 5.9	70.9 ± 6.0	10/18	PBMCs	Autologous	i.m.	3 × 10^9^/leg	12 weeks
Zhang et al. (2007)	China	RCT	61	NR		65 ± 7	63 ± 6	36/25	BMSCs	Autologous	i.m.	NR	40 days
Debin et al. (2008)	China	RCT	45	4.2 ± 1.0	3.8 ± 1.1	64.3 ± 12.7		19/26	BMMSCs	Autologous	i.m.	7.32 × 10^8^ to 5.61 × 10^9^/leg	12 weeks
Han et al. (2010)	Italy	RCT	52	4.3 ± 2.1	4.0 ± 2.1	66.5 ± 7.5	68.4 ± 8.7	23/29	PLA cells	Autologous	Ad.us.ext	>4 × 10^6^/ulcer	8 Weeks
Jain et al. (2011)	India	RCT	48	NR		(33,76)	(28,69)	17/31	BMDCs	Autologous	NA		12 weeks
Lu et al. (2011)	China	RCT	37	NR		63 ± 8	65 ± 10	22/15	BMMSCs/BMMNCs	Autologous	i.m.	9.6 × 10^8^/leg; 9.3 × 10^8^/leg	24 Weeks
Ozturk et al. (2012)	Turkey	RCT	40	NR		71.9 ± 9.2	70.8 ± 8.8	11/29	PBMCs	Autologous	i.m.	2.48 × 10^7^/leg	12 weeks
Kirana et al. (2012)	Germany	RCT	24	9.6 ± 4.2	NA	68.5 ± 1.5	70.9 ± 1.7	5/19	BMMNCs/BMTRCs	Autologous	i.m. or i.a.	3 × 10^8^/leg; 8 × 10^7^/leg	45 weeks
You et al. (2012)	Korea	RCT	46	4.0 ± 3.5	5.2 ± 6.4	63.5 ± 9.0	62.4 ± 9.4	14/32	Keratinocytes	Allogenic	Dressing	NR	12 weeks
Dubsky et al. (2013)	Czech Republic	CCT	50	5.2 ± 1.6	5.9 ± 2.0	61.8 ± 9.8	63.3 ± 9.1	9/41	PBPCs/BMMNCs	Autologous	i.m.	2.2 × 10^9^/leg; 2.4 × 10^10^/leg	6 months
Mohammadzadeh et al. (2013)	Iran	RCT	21	15.8 ± 17.0	14.2 ± 4.1	63.5 ± 7.8	64.2 ± 7.8	NA	PBMSCs	Autologous	i.m.	(9.0–12.0)×10^8^/leg	3 months
Dubsky et al. (2014)	Czech Republic	CCT	54	NR		62.7 ± 10.4	62.7 ± 9.1	11/43	BMMNCs/PBPCs	Autologous	i.m.	NR	12 months
Lavery et al. (2014)	United States	RCT	97	3.4 ± 3.2	3.9 ± 3.2	55.5 ± 11.5	55.1 ± 12.0	29/68	hVWM	Allogenic	Dressing	NR	84 days
He (2014)	China	RCT	100	NR		63.3	63.2	46/54	HUCMSCs	Autologous	i.m.	(5.8–8.2)×10^7^/leg	3 months
Zhang et al. (2016)	China	CCT	53	NR		71.3 ± 9.1	71.6 ± 9.1	26/27	CD133+ cells	Autologous	i.a.	≥1 × 10^7^/leg	18 months
Park et al. (2018)	Korea	RCT	167	2.8 ± 3.7	2.4 ± 2.7	56.5 ± 12.7	59.3 ± 12.6	63/104	rhEGF	Allogenic	Spray	50 μg/mL	12 weeks
Lonardi et al. (2019)	Italy	RCT	114	NR		69.0 ± 11.6	71.6 ± 10.8	28/86	MFAT	Autologous	i.r.w.	10–30 mL/leg	6 months
Moon et al. (2019)	Korea	RCT	39	2.0 ± 0.9	2.8 ± 2.0	59.9 ± 13.3	68.4 ± 9.9	12/27	ADSCs	Allogenic	Dressing	1 × 10^6^ cells/sheet	12 weeks
Smith et al. (2020)	United Kingdom	RCT	12	6.3 ± 2.6	7.0 ± 4.5	60.2	55.2	2/10	ADSCs	Autologous	Dressing	NR	12 weeks
Uzun et al. (2021)	Turkey	RCT	20	23.5 ± 5.6	25.8 ± 5.4	57.5 ± 8.4	57.2 ± 4.5	8/12	ADSCs	Allogenic	i.m.	6 × 10^6^ cells/leg	48 months
Liu et al. (2023b)	China	RCT	100	NR		65.9 ± 6.2	64.1 ± 6.1	34/66	BMSCs	Autologous	i.m.	NR	30 days
Mrozikiewicz-Rakowska et al. (2023)	Poland	CCT	46	2.7 ± 2.9	2.7 ± 1.6	56.7 ± 11.1	61.7 ± 7.5	9/37	ADSCs	Allogenic	Dressing	NR	49 days
Wang et al. (2024a)	China	RCT	43	9.7 ± 4.9	10.2 ± 5.2	61		15/28	PBSCs	Autologous	i.m.	2 × 10^6^/ulcer	12 weeks
Wang et al. (2024b)	China	RCT	86	NR		63.1 ± 6.4	62.8 ± 6.4	33/53	UCMSCs	Allogenic	i.m.	1 × 10^7^ cells/sheet	5 weeks

RCT, randomized controlled trial; CCT, clinical controlled trials; NR, no reported; PBMCs, peripheral blood mononuclear cells; i.m., intramuscular injection; i.a., intra-arterial injection; i.r.w., injected radially into wound; BMSCs, bone marrow stem cells; PLA, human processed lipoaspirate; Ad.us.ext, ad usum externum (for external use); BMMNCs, bone marrow mesenchymal stem cells; BMMNCs, bone marrow-derived mononuclear cells; PBPCs, peripheral blood progenitor cells; PBMSCs, peripheral blood mesenchymal stem cells; hVWM, human viable wound matrix; rhEGF, recombinant human epidermal growth factor; MFAT, micro-fragmented adipose tissue; ADSCs, adipose-derived stem cells; UCMSCs, umbilical cord mesenchymal stem cells.

### Quality assessment

**FIGURE 2:**
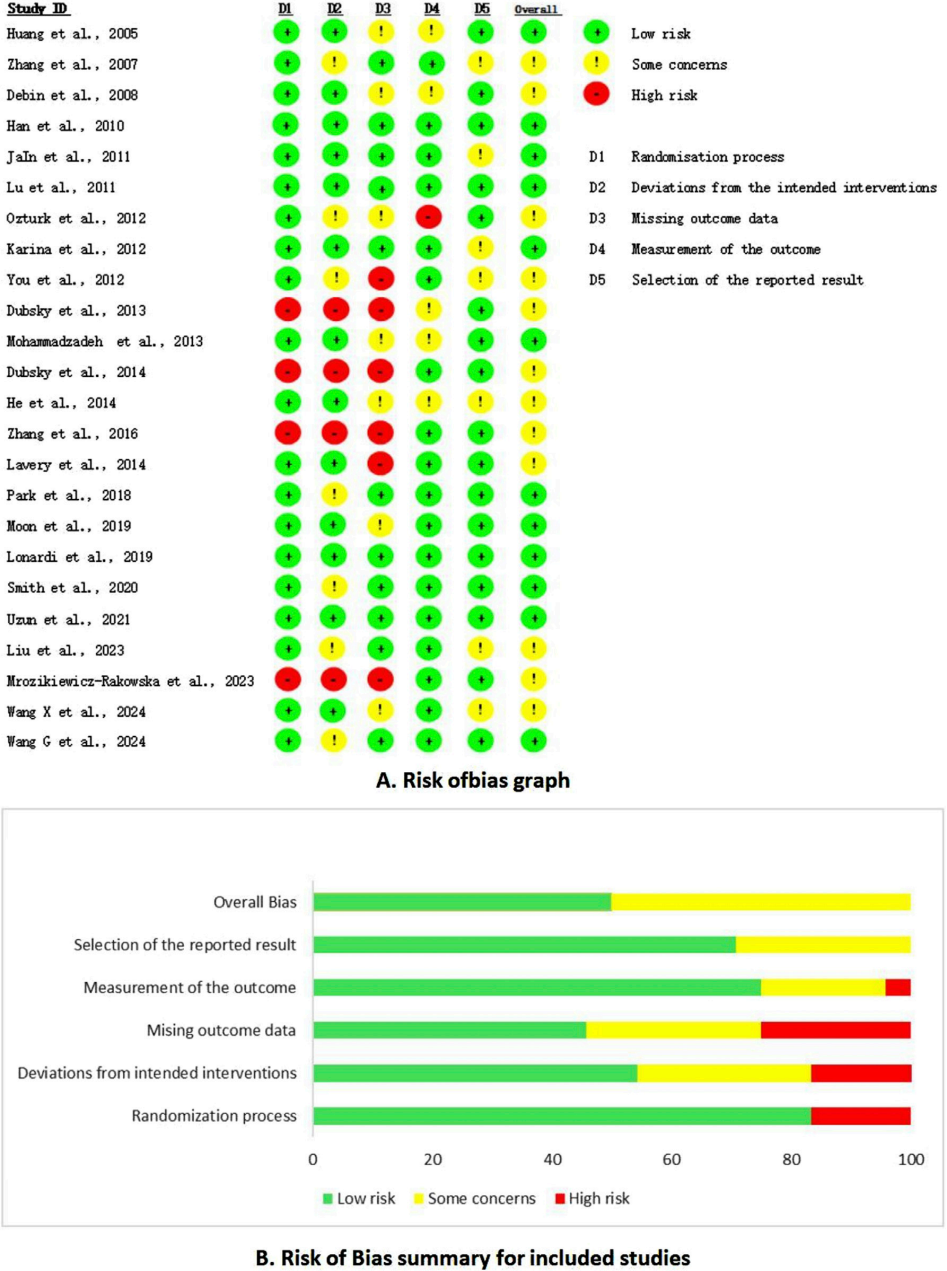
Risk of bias summary. **(A)** Risk of bias graph. **(B)** Risk of bias summary for included studies.

A thorough quality assessment revealed that 20 studies provided detailed descriptions of the randomization methods employed. However, seven studies, despite mentioning randomization, failed to elucidate the specific methods utilized. The risk of bias was categorized as low in twelve articles, with the remaining articles falling into the medium-risk category; notably, no studies were classified as high risk (Figure 2).

### Wound healing rate by cell sources

**FIGURE 3:**
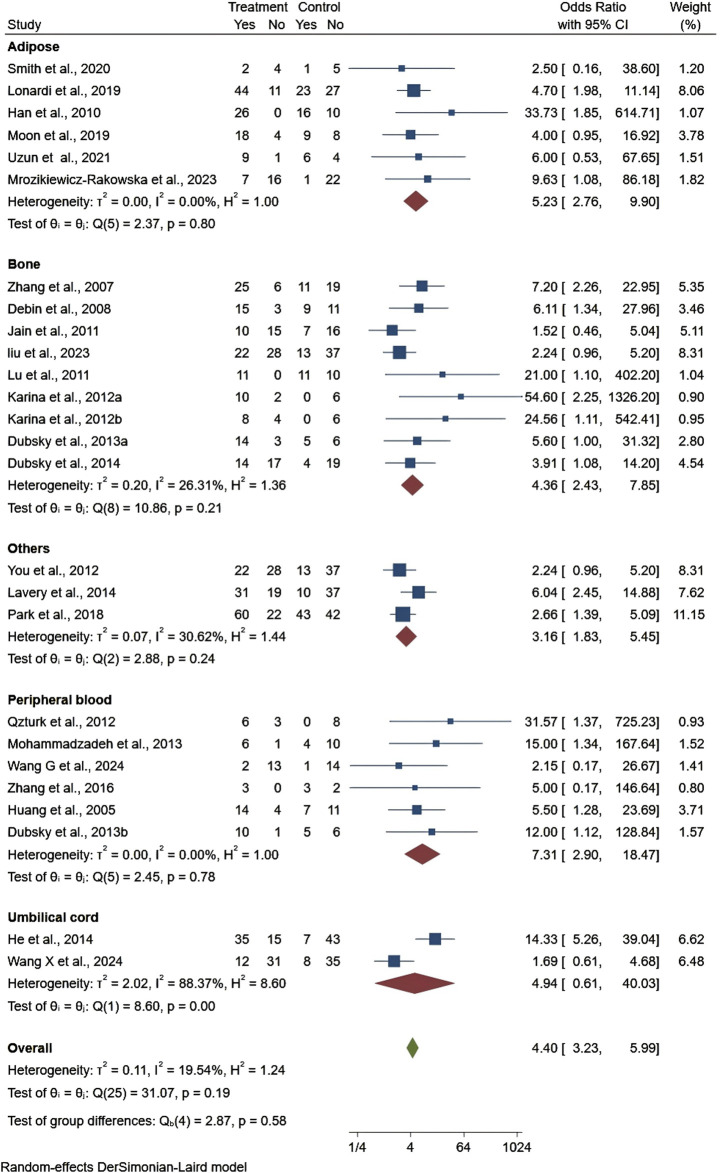
Forest plot showing the effect of stem cells from different sources on ulcer healing rates.

The healing rate of wounds treated with stem cells from different sources showed obvious improvement, with peripheral blood-derived stem cells demonstrating the highest efficacy (OR = 7.31, 95% CI: 2.90–18.47). Adipose-derived stem cells were the second most effective (OR = 5.23, 95% CI: 2.76–9.90), followed by umbilical cord-derived stem cells (OR = 4.94, 95% CI: 0.61–40.03), bone marrow-derived stem cells (OR = 4.36, 95% CI: 2.43–7.85), and stem cells from other sources (OR = 3.16, 95% CI: 1.83–5.45). The heterogeneity ranged from non-existent in the adipose and peripheral blood groups (I^2^ = 0.00%) to moderate in the bone groups (I^2^ = 26.31%) and other groups (I^2^ = 30.62%), and substantial in the umbilical cord groups (I^2^ = 88.37%). Nevertheless, only umbilical cord-derived stem cells showed statistical significance (p < 0.05) (Figure 3). The L’Abbé plot analysis shows high consistency and low heterogeneity across stem cells from different sources in promoting DFU healing, with most studies aligning closely with the equality line. However, some studies deviated, indicating that certain stem cell sources may exhibit stronger or weaker effects under specific conditions. (Supplementary Figure S1).

### Publication bias

In the funnel plots of studies on different sources of stem cells, only studies on bone derived stem cells showed uneven distribution, indicating potential bias (Supplementary Figure S2). The overall funnel plot shows asymmetry, indicating possible publication bias in the overall analysis (Supplementary Figure S3). Both Begg’s and Egger’s tests suggested the possibility of potential publication bias (P < 0.01). To address this, we performed a trim-and-fill analysis. After adjusting for the imputed studies, the pooled OR decreased from 4.40 (95% CI: 3.23–5.99) to 3.40 (95% CI: 2.39–4.84).

### Sensitivity analyses

The included literature demonstrated that excluding each study in turn had no impact on the results (Supplementary Figure S4).

### Subgroup analysis

The subgroup analysis investigated the effects of stem cell therapy on wound healing rates in diabetic foot ulcers, stratifying the data by follow-up duration, study design, and donor type (Table 2). In the group with a follow-up time of 3 months or less, peripheral blood stem cells demonstrated the most favorable therapeutic effect (OR = 7.54, 95% CI: 2.88–19.77), though it did not reach statistical significance (P > 0.05), while umbilical cord stem cell therapy had statistically significant efficacy (P < 0.05), despite higher heterogeneity (I^2^ = 88.37%). In the group with a follow-up duration of more than 3 months, bone marrow-derived stem cells showed the best therapeutic effect (OR = 29.72, 95% CI: 5.04–175.37). Regarding study design, both CCT and RCT studies demonstrated the effectiveness of peripheral blood, bone marrow, and adipose-derived stem cells in treating diabetic foot, with higher heterogeneity observed in the RCT group. Analysis by donor type showed that allogenic stem cells, particularly adipose-derived stem cells (OR = 5.36, 95% CI: 1.82–15.77), also had favorable therapeutic effects for diabetic foot. In autologous stem cell group, peripheral blood-derived stem cells demonstrated the most favorable therapeutic effect compared to bone-derived and adipose-derived stem cells (OR = 7.31, 95% CI: 2.90–18.47).

**TABLE 2:** The results of subgroup analysis.

	n	Odds ratio	95% CI	I^2^, %	P value
Follow-up duration					
Less than or equal to 3 months					
Peripheral blood	5	7.54	2.88–19.77	0.00	0.66
Bone	6	3.36	2.03–5.53	4.17	0.39
Adipose	3	4.63	1.54–13.94	0.00	0.72
Umbilical cord	2	4.94	0.61–40.03	88.37	<0.05
Others	3	3.16	1.83–5.45	30.62	0.24
More than 3 months					
Peripheral blood	1	5.00	0.17–146.64	—	—
Bone	3	29.72	5.04–175.37	0.00	0.90
Adipose	3	5.56	2.54–12.17	0.00	0.44
Study design					
CCT					
Peripheral blood	1	12.00	1.12–128.84	—	—
Bone	2	4.45	1.59–12.49	0.00	0.74
Adipose	1	9.63	1.08–86.18	—	—
RCT					
Peripheral blood	5	7.99	1.99–32.01	0.00	0.55
Bone	7	4.82	2.17–10.70	43.74	0.10
Adipose	5	4.94	2.53–9.63	0.00	0.73
Umbilical cord	2	4.94	0.61–40.03	88.37	<0.05
Others	3	3.16	1.83–5.45	30.62	0.24
Donor type					
Allogenic					
Adipose	3	5.36	1.82–15.77	0.00	0.80
Others	3	3.16	1.83–5.45	30.62	0.24
Umbilical cord	2	4.94	0.61–40.03	88.37	<0.05
Autologous					
Peripheral blood	6	7.31	2.90–18.47	0.00	0.78
Bone	9	4.36	2.43–7.85	26.31	0.21
Adipose	3	5.16	2.34–11.39	0.00	0.38

RCT, randomized controlled trial; CCT, clinical controlled trials.

### Secondary outcomes

**FIGURE 4:**
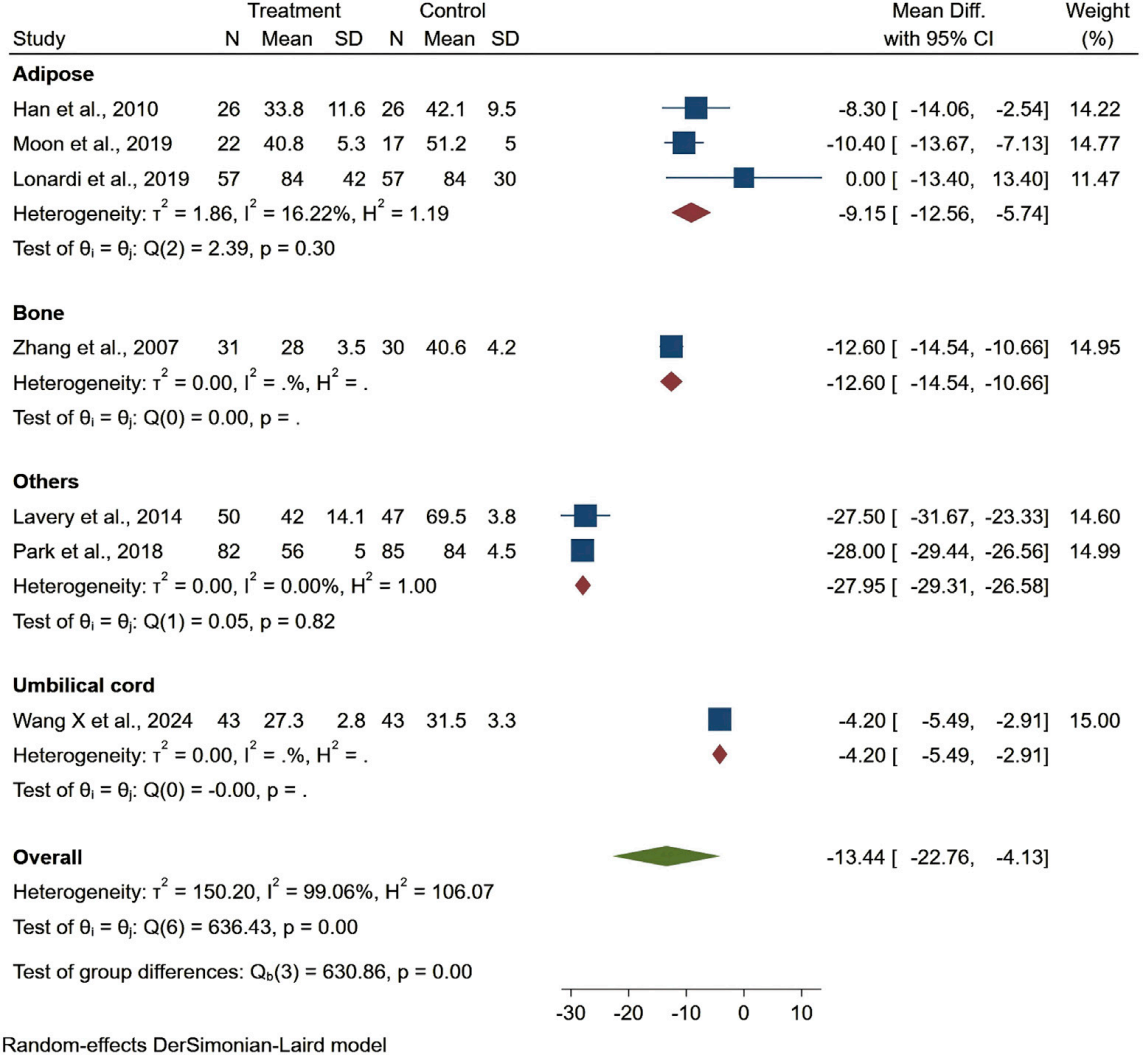
Forest plot showing the effect of stem cells from different sources on ulcer healing time.

**FIGURE 5:**
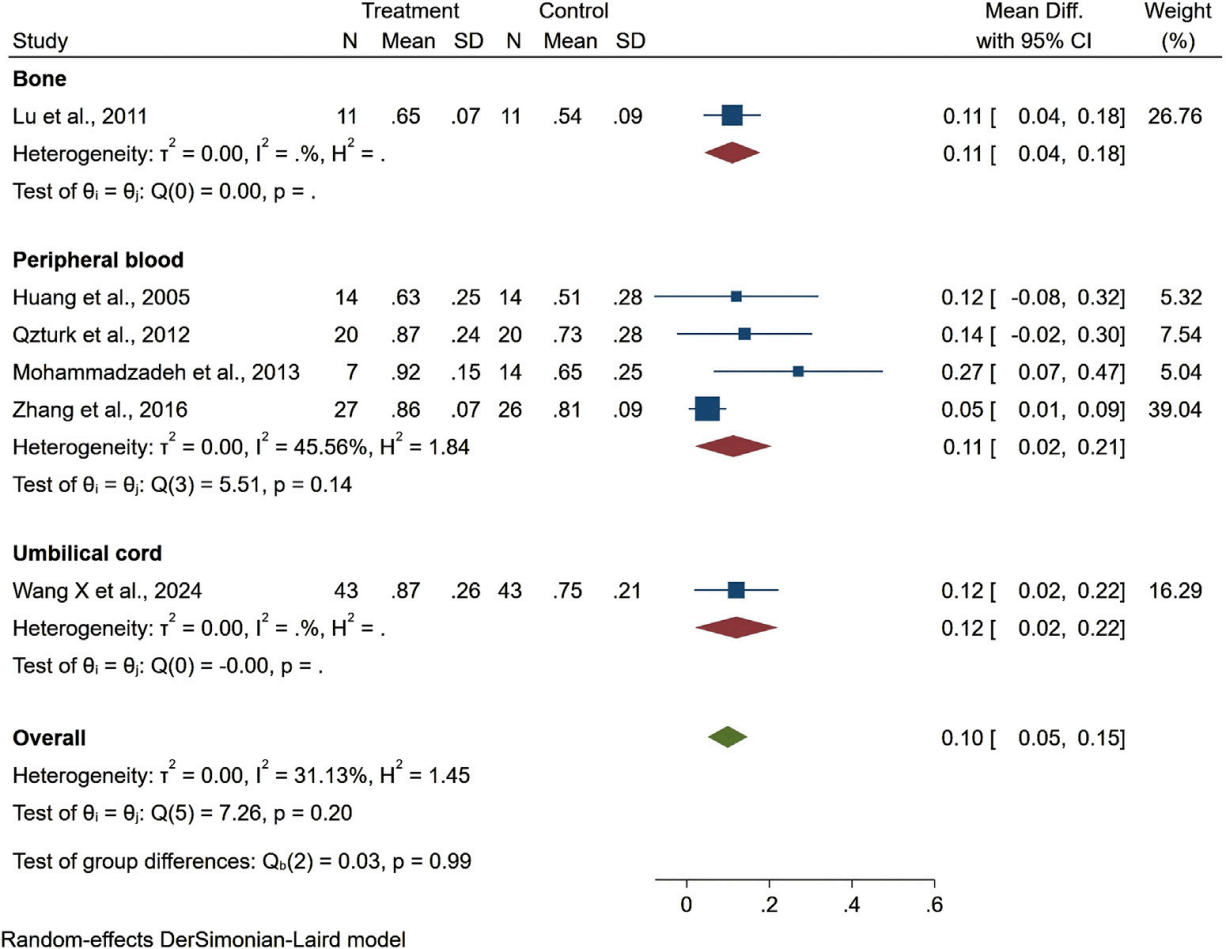
Forest plot showing the effect of stem cells from different sources on ABI.

**FIGURE 6:**
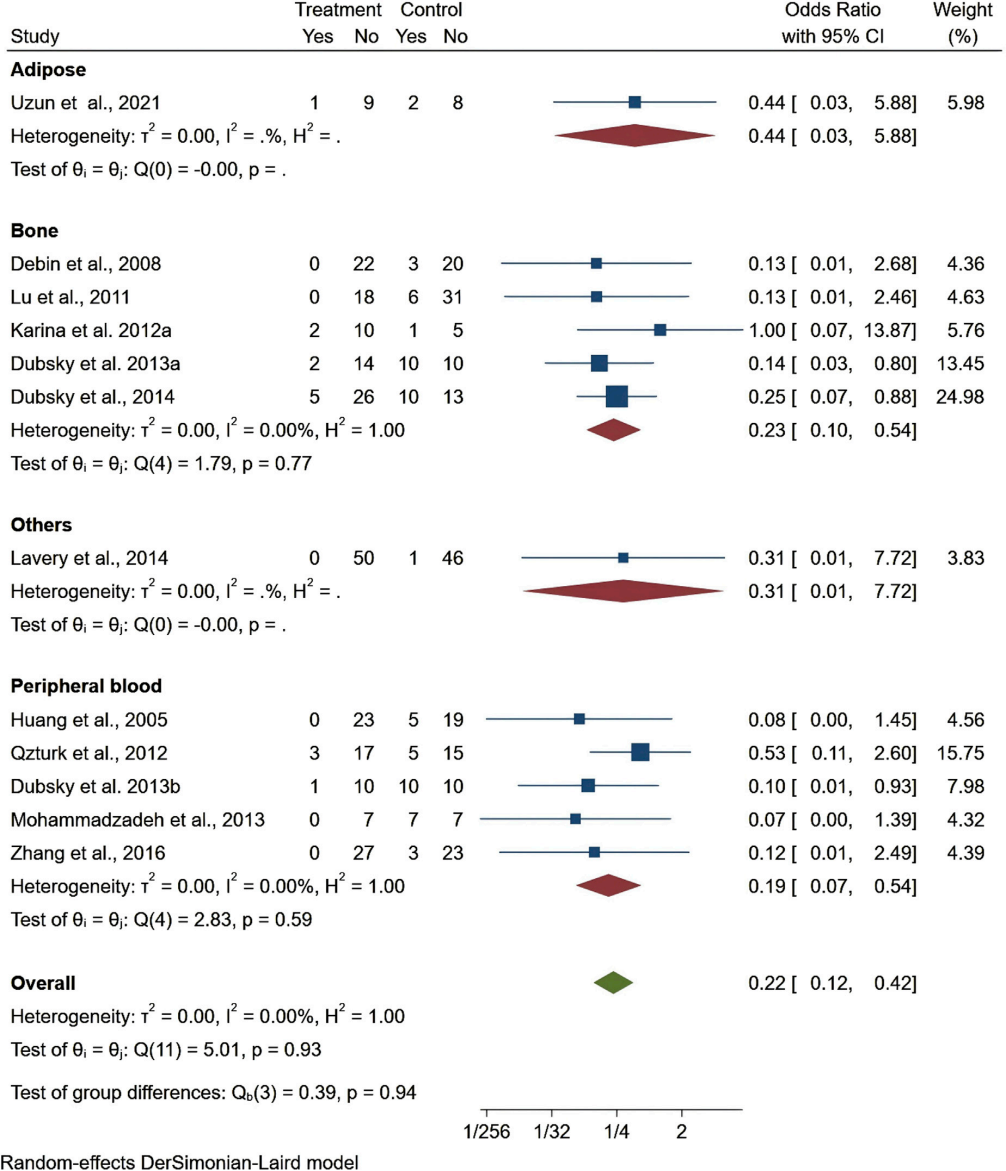
Forest plot showing the effect of stem cells from different sources on amputation rates.

Seven studies reported healing time, highlighting 13.44 days shorter of the trial group than the control one (95% CI −22.76 to −4.13, P < 0.01). Among them, patients treated with stem cells from other sources had significantly shorter ulcer healing times compared to those treated with bone marrow, adipose tissue, or umbilical cord stem cells. However, the results for ulcer healing time showed considerable heterogeneity (I^2^ = 99.06%), but the overall result was statistically significant (Figure 4). Regarding ankle brachial index (ABI), the results showed a slight improvement (MD = 0.10, 95% CI: 0.05–0.15), but did not reach statistical significance (P > 0.05) (Figure 5). Although the results for amputation rate also did not reach statistical significance (P > 0.05), all stem cell sources demonstrated consistent positive effects (OR = 0.22, 95% CI: 0.12–0.42) (Figure 6).

## Discussion

This study assessed the efficacy of various stem cell therapies in promoting ulcer healing in patients with DFU. The efficacy varied across different stem cell sources, with peripheral blood-derived cells demonstrating the most potent effect, while adipose-derived, umbilical cord-derived, bone marrow-derived, and other source stem cells also contributed to improved ulcer healing. Heterogeneity across the studies was relatively moderate, suggesting a reasonable consistency among the included studies despite the varied therapeutic outcomes. However, publication bias, as indicated by funnel plot asymmetry and confirmed through the trim-and-fill method, suggested an initial overestimation of treatment effects. Subgroup analysis revealed that treatment outcomes were influenced by follow-up duration, study design, and donor type, although no significant statistical differences were found between the groups except for umbilical cord derived stem cells. Additionally, stem cell therapy demonstrated positive clinical effects in accelerating wound healing, improving ABI, and reducing amputation rates, further highlighting its potential in the management of diabetic foot ulcers.

In recent years, numerous studies have explored the potential efficacy of stem cells in treating diabetic foot ulcers, examining their specific molecular mechanisms, types, and expanded applications, with the goal of advancing the clinical use of stem cell therapy for DFU (Galindo et al., 2011; Xia et al., 2024). Previous studies demonstrated a superior therapeutic impact of cellular interventions, assessing not only therapeutic efficacy but also the critical outcome of amputation rates, although the studies were limited by small sample sizes and a lack of variability in outcome measures (Guo et al., 2017; Dai et al., 2020). More recently, Sun et al., in 2022 analyzed fourteen studies with a total of 683 participants, revealing that cell-based therapy improved several outcomes including wound healing rates, vascular neogenesis in the lower extremities, TcPO2, ABI values, and pain-free walking distances. Additionally, these interventions were linked to reduced amputation rates and resting pain scores. These results are consistent with those of the current study, particularly in terms of vascular neogenesis and improvements in pain management and mobility. However, significant gaps remain in the research on TcPO2, pain-free ambulation, and rest-induced pain, highlighting the need for more comprehensive studies. The analysis also pointed out the lack of subgroup analyses that could differentiate the effects of various cellular therapies, adding a layer of uncertainty to the results (Sun et al., 2022a).

DFU is a prevalent and severe complication of diabetes, impacting skin, muscle, nerve, and vascular systems, thereby complicating the healing process (Sun et al., 2022b). The pathogenesis is driven by a high-sugar microenvironment that elevates oxidative stress, impairing wound healing (Patel et al., 2019). This environment also facilitates reactions between proteins and glucose, producing harmful byproducts that inhibit cell proliferation and adversely affect vascular and neural functions (Davis et al., 2020). Furthermore, an environment rich in fats and glucose promotes inflammatory responses, reducing neutrophil chemotaxis and macrophage efficacy, and hindering the macrophage phenotype shift from M1 to M2 (Boniakowski et al., 2019; Parisi et al., 2018). These complex mechanisms contribute to the high recurrence and worsening of DFU, placing significant psychological and economic burdens on patients. Addressing these challenges is critical and enhancing the clinical cure rate and accelerating wound healing are priorities in diabetes research. While it has been noted that VEGF and fibroblast growth factor levels are comparable in peripheral and bone marrow-derived cells, levels of interleukin-1b and tumor necrosis factor are higher in peripheral blood, suggesting less efficacy of peripheral blood mononuclear cells compared to bone marrow-derived cells (Iba et al., 2002).

Back to our findings, Peripheral Blood-derived stem cells might be particularly effective due to their high availability and the potential presence of a wide range of progenitor cells, which can contribute to enhanced angiogenesis and tissue regeneration (Rai et al., 2022). Adipose-derived stem cells, while also effective, contain a rich mix of regenerative factors and have been shown to promote wound healing through their anti-inflammatory properties and ability to enhance collagen deposition (Zhang et al., 2020). Bone-derived stem cells are well-noted for their osteogenic and chondrogenic potential but may offer slightly less efficacy in soft tissue regeneration compared to other cell sources (Polymeri et al., 2016). Umbilical cord-derived stem cells, due to their unique regenerative capabilities, including anti-inflammatory, immunomodulatory, and tissue repair properties, show potential in the treatment of diabetic foot ulcers; however, the lack of standardized treatment protocols may result in variability in their efficacy (Yu et al., 2023). These findings underscore the importance of selecting an appropriate stem cell source based on the specific clinical aspects and healing requirements of diabetic foot ulcers. The choice of stem cell type should consider the wound environment, the patient’s overall health status, and the specific healing mechanisms needed (Lopes et al., 2018).

In addition, although our study found that peripheral blood derived stem cells and bone derived stem cells showed the most favorable therapeutic effects at follow-up times of 3 months or less and 3 months or more, respectively, the small overall sample size may be an important influencing factor. For example, a 12-week trial in Turkey used autologous peripheral blood mononuclear cell transplantation to treat DFU, with a cure rate 31 times higher than that of patients receiving standard treatment. However, the total number of patients was only 40 (Ozturk et al., 2012). Another study evaluated the effects of autologous bone marrow mononuclear cells and CD90^+^ enriched tissue repair cells in treating DFU patients. The treatment effects of the two cell on ulcer healing rates were 54 times and 25 times higher than that of the control group, respectively, but the total sample size was only 24 (Kirana et al., 2012). In contrast, Lonardi et al.’s study included over 100 patients who received adipose-derived stem cell therapy, but the treatment effect in the intervention group was only four times higher than that of the control group (Lonardi et al., 2019). Additionally, we observed that umbilical cord-derived stem cells had a significant effect on wound healing rates. One study using umbilical cord mesenchymal stem cell transplantation in DFU patients found that the observation group had significantly shorter healing times and faster wound closure compared to the control group, with the healing rate approximately 14 times higher (He, 2014). These findings emphasize the need for more and larger sample size studies to confirm the potential of stem cells from various sources in treating DFU.

However, there are some limitations to our meta-analysis. First, the relatively small sample sizes may limit the applicability of our findings to larger, more diverse patient populations. Second, the inherent heterogeneity among the included studies, due to variations in stem cell sources, protocols, wound environments, and patient demographics, could affect the generalizability of the findings. Although we employed a random-effects model to address this variability, the differences in study designs and treatment regimens across studies still pose a challenge in interpreting the pooled results uniformly. Third, the presence of publication bias, as indicated by the asymmetry in funnel plots and confirmed by the trim-and-fill method, suggests that smaller studies with negative results might be underrepresented in the literature, potentially skewing the efficacy estimates. Lastly, the quantitative analysis was limited to published studies; thus, unpublished data and ongoing research could alter the effectiveness and safety profiles presented.

Future research should prioritize understanding the mechanisms behind stem cell-mediated wound healing and how patient-specific factors like genetics and metabolic control influence treatment outcomes. Comparative studies are essential to determine the most effective sources and types of stem cells, while the development of standardized protocols for cell preparation and application will enhance treatment consistency and efficacy. Longitudinal studies should evaluate the long-term effects of therapy, and integration with other treatments could lead to comprehensive care strategies. Additionally, addressing regulatory, ethical, and economic issues will be crucial for facilitating clinical implementation and ensuring the therapy’s cost-effectiveness compared to conventional treatments.

## Conclusion

This study shows that stem cell therapy may be a promising method to promote wound healing in patients with diabetes foot ulcers, but its effectiveness varies depending on the source of stem cells. Despite evidence of moderate heterogeneity and publication bias, this analysis demonstrates the overall effectiveness of stem cell therapy in improving clinical outcomes in DFU management.

## Supplementary material

SUPPLEMENTARY FIGURE S1 L'Abbé plot for trials evaluating the impact of stem cells from different sources on the healing rate in DFU.

SUPPLEMENTARY FIGURE S2 Funnel plot of stem cells from different sources on the healing rate in DFU.

SUPPLEMENTARY FIGURE S3 Overall Funnel plot of stem cells on the healing rate in DFU.

SUPPLEMENTARY FIGURE S4 Sensitivity analysis of stem cells from different sources on the healing rate in DFU.
