# Supplementary figures and images for: Impacts of stem cells from different sources on wound healing rate in diabetic foot ulcers: a systematic review and meta-analysis
Source: Front Genet. 2025 Jan 28;15:1541992. doi: 10.3389/fgene.2024.1541992 (PMC11811113; doi:10.3389/fgene.2024.1541992)

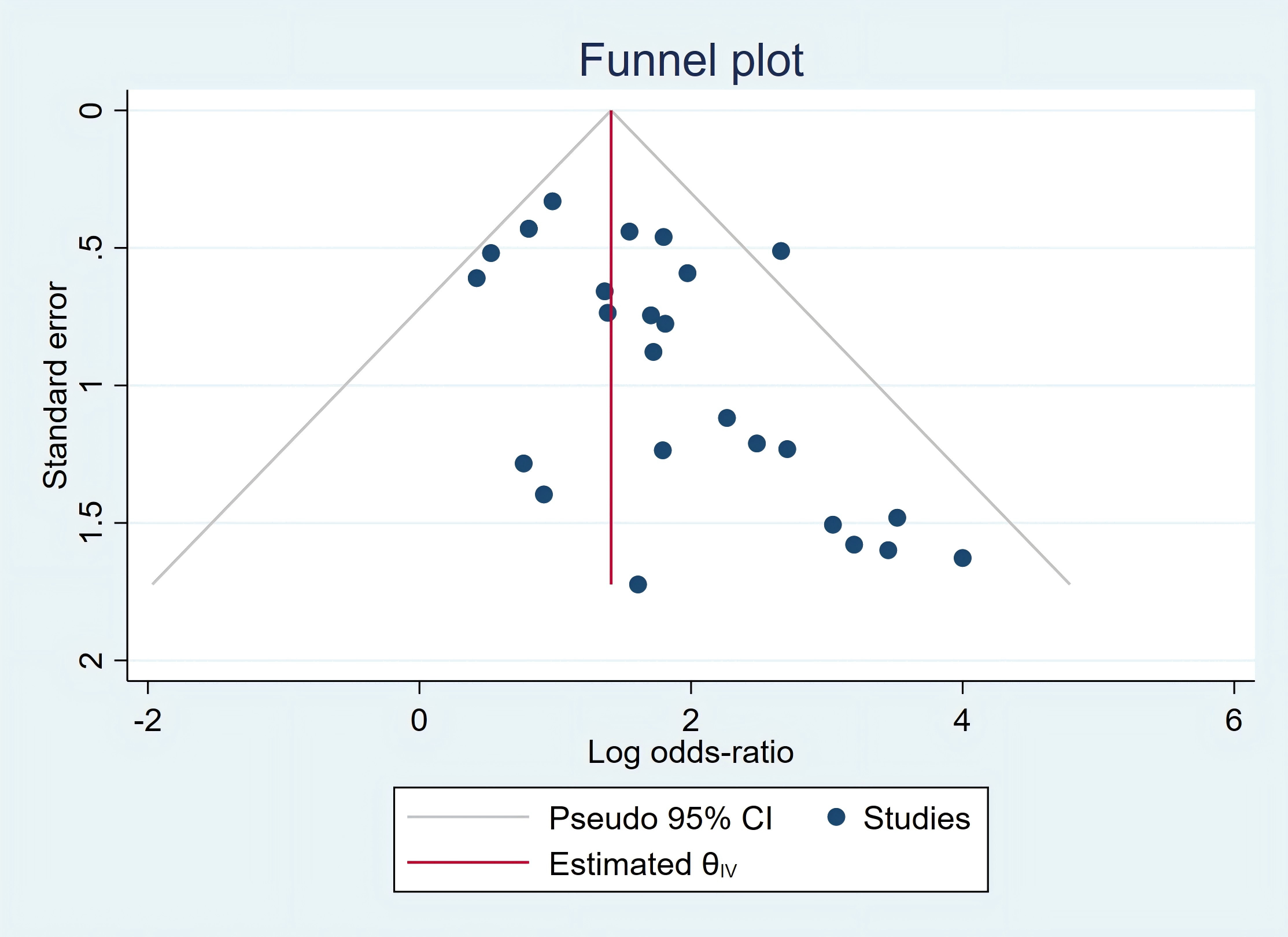

Supplement: Supplementary file 1 [file Image3.tiff]

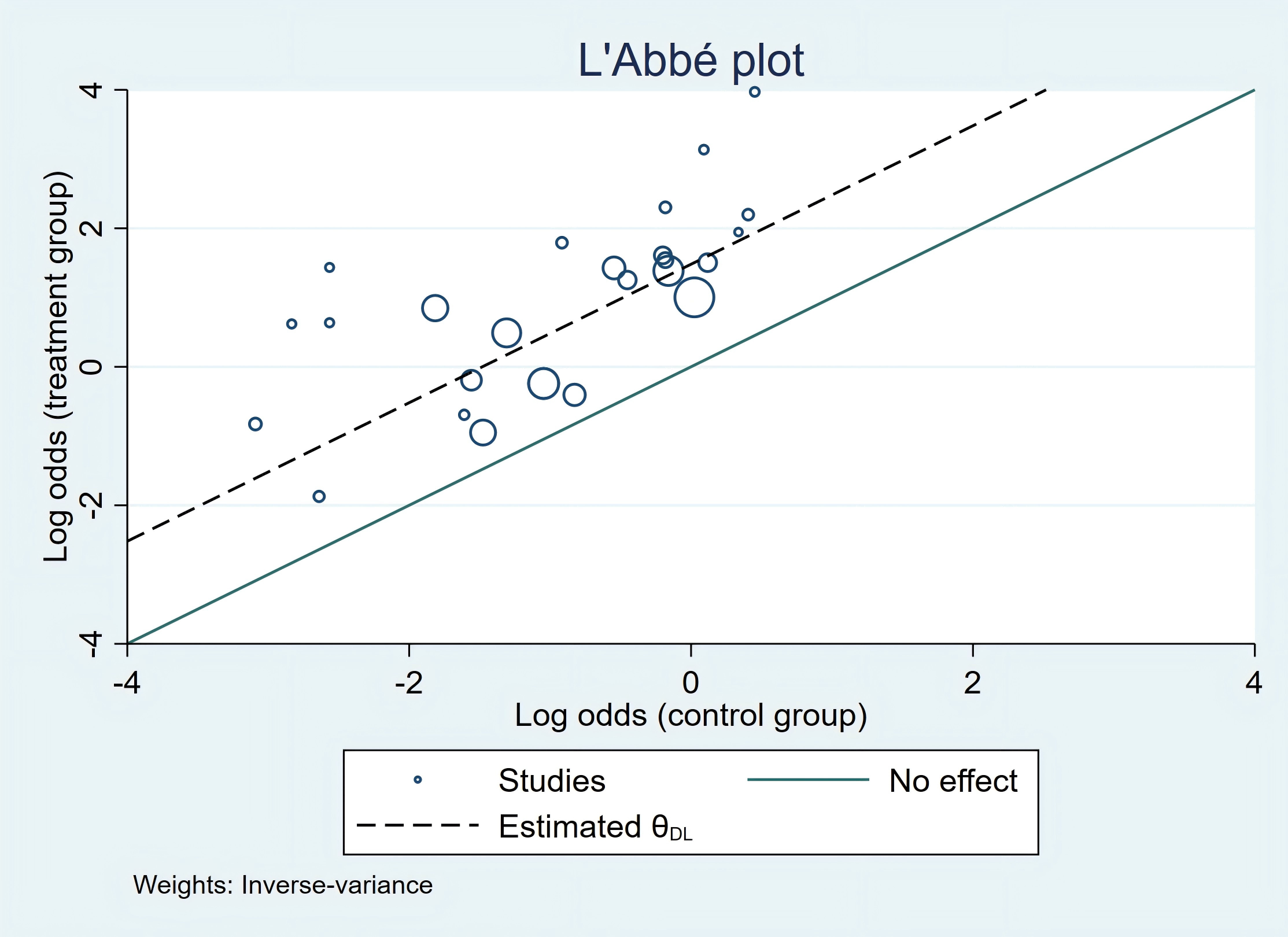

Supplement: Supplementary file 2 [file Image1.tiff]

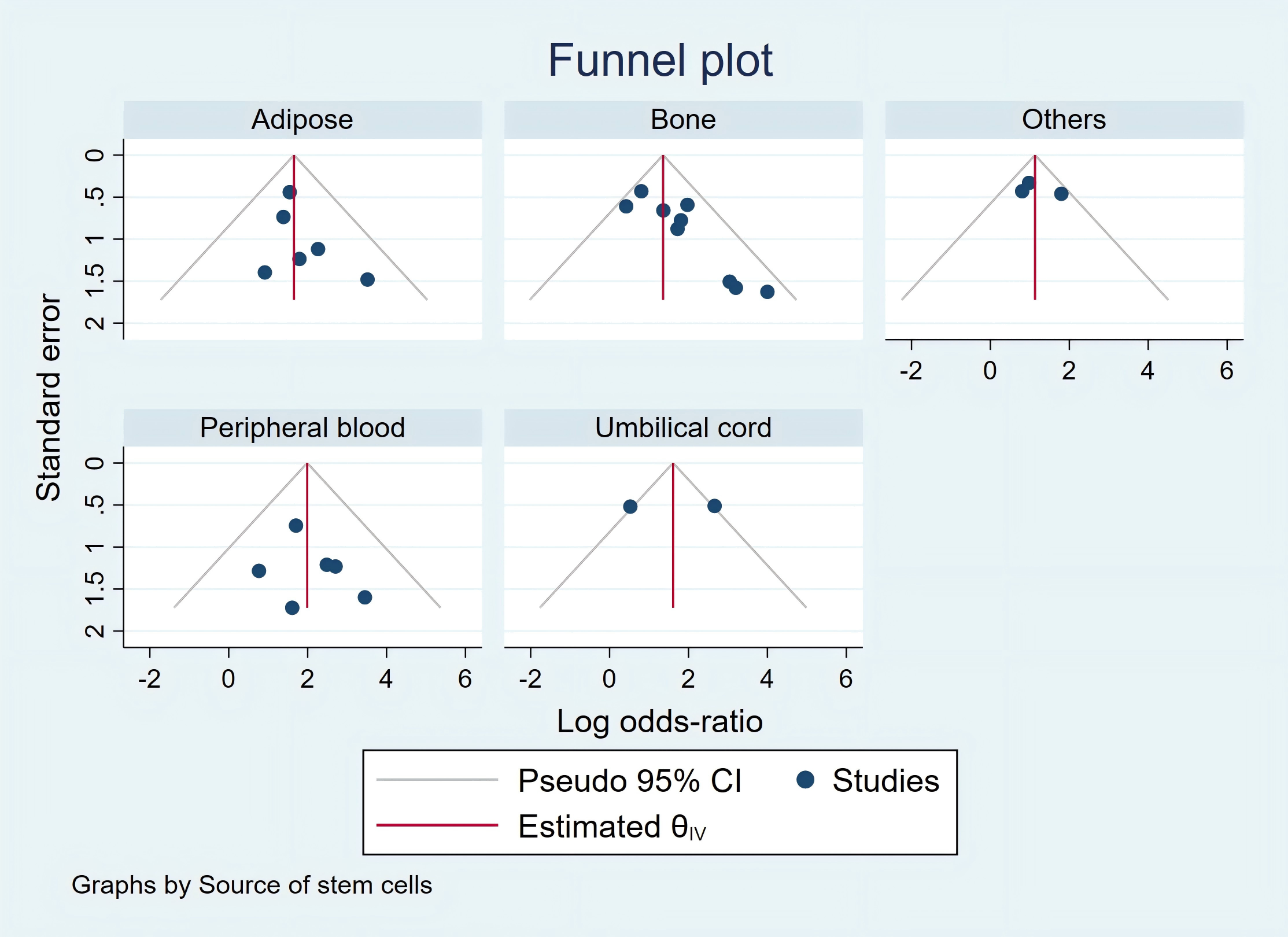

Supplement: Supplementary file 4 [file Image2.tiff]

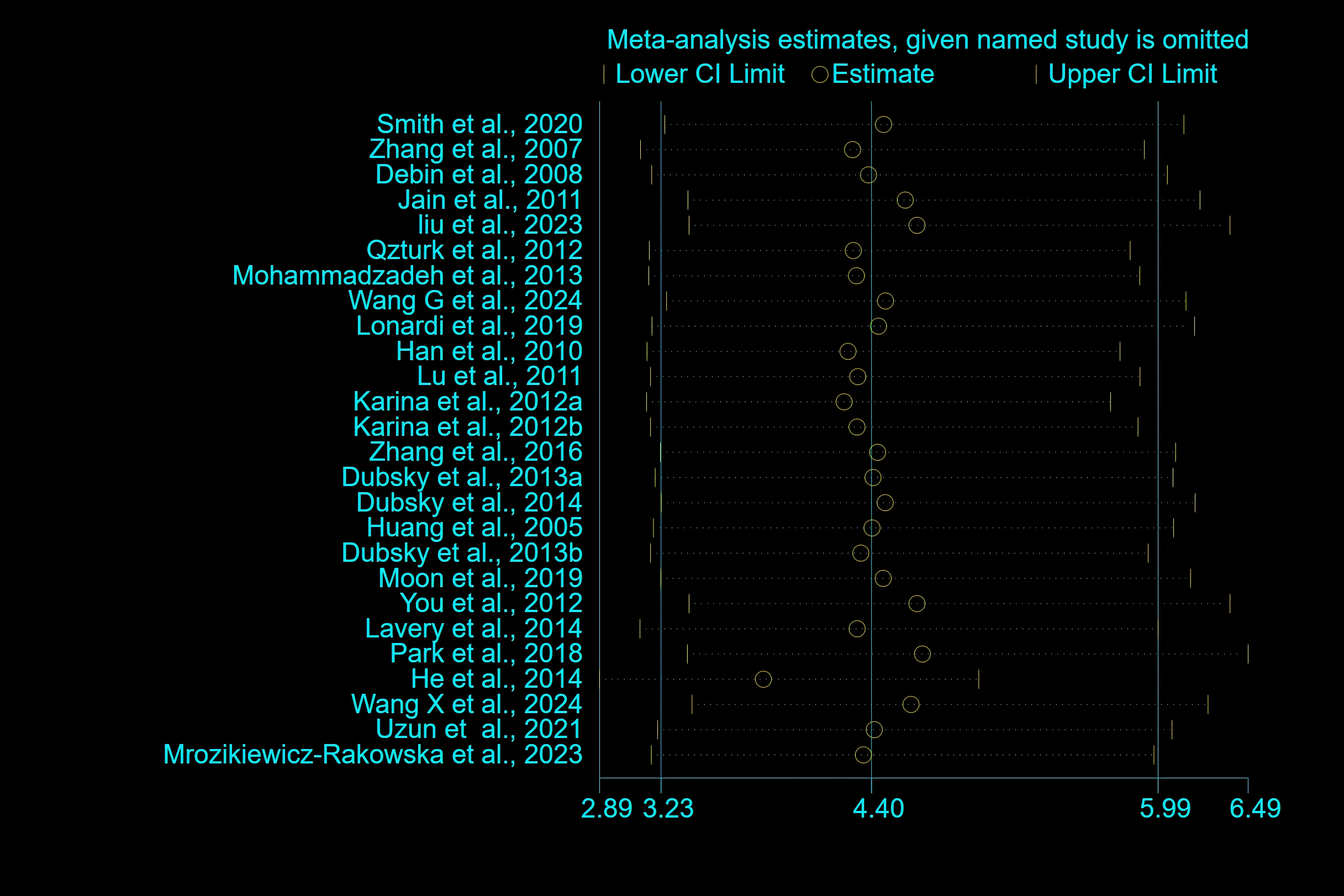

Supplement: Supplementary file 5 [file Image4.tiff]
