# Supplementary material for: Impacts of stem cells from different sources on wound healing rate in diabetic foot ulcers: a systematic review and meta-analysis
Source: Front Genet. 2025 Jan 28;15:1541992. doi: 10.3389/fgene.2024.1541992 (PMC11811113; doi:10.3389/fgene.2024.1541992)
Supplement: Supplementary file 3 [file Table1.docx]

# Table S1. Search Strategy

| Database | Search strategy |
| --- | --- |
| PubMed | (("stem cell" OR "progenitor cell" OR "mesenchymal stem cells" OR "adipose-derived" OR "bone marrow" OR "peripheral blood" OR "Umbilical cord" OR "mononuclear cell") AND ("diabetic") AND ("foot" OR "ulcer" OR "wound") ) in All Fields |
| Web of Science | (("stem cell" OR "progenitor cell" OR "mesenchymal stem cells" OR "adipose-derived" OR "bone marrow" OR "peripheral blood" OR "Umbilical cord" OR "mononuclear cell") AND ("diabetic") AND ("foot" OR "ulcer" OR "wound") ) in All Fields |
| Cochrane Library | (("stem cell" OR "progenitor cell" OR "mesenchymal stem cells" OR "adipose-derived" OR "bone marrow" OR "peripheral blood" OR "Umbilical cord" OR "mononuclear cell") AND ("diabetic") AND ("foot" OR "ulcer" OR "wound") ) in All Text |
| CNKI | "stem cell"[All Text] AND "diabetes"[All Text] OR " foot" [All Text] OR "ulcer"[All Text] |
| Wanfang | "stem cell"[All Text] AND "diabetes foot ulcer"[All Fields] |
| Embase | ('Stem Cell Transplantation'/exp OR 'Mesenchymal Stem Cells'/exp OR 'Adipose-Derived Stem Cells'/exp OR 'Bone Marrow Stem Cells'/exp OR 'Peripheral Blood Stem Cells'/exp OR 'Umbilical Cord Stem Cells'/exp OR 'Mononuclear Cell'/exp) AND ('Diabetes Mellitus'/exp OR 'Diabetic'/exp) AND ('Foot Ulcer'/exp OR 'Diabetic Foot Ulcer'/exp OR 'Wound'/exp OR 'Diabetic Foot'/exp) |
